# Impact of Clarified Apple Juices with Different Processing Methods on Gut Microbiota and Metabolomics of Rats

**DOI:** 10.3390/nu14173488

**Published:** 2022-08-25

**Authors:** Lei Xu, Shini Yang, Kewen Wang, Anjing Lu, Xue Wang, Zhenzhen Xu

**Affiliations:** 1 Institute of Quality Standard & Testing Technology for Agro-Products, Chinese Academy of Agricultural Sciences, Key Laboratory of Agro-Food Safety and Quality, Ministry of Agriculture and Rural Affairs, Beijing 100081, China; 2 College of Food Science and Nutritional Engineering, China Agricultural University, Beijing Key Laboratory for Food Nonthermal Processing, Key Lab of Fruit and Vegetable Processing, Ministry of Agriculture and Rural Affairs, Beijing 100083, China; 3 China Center for Information Industry Development, Beijing 100048, China

**Keywords:** clarified apple juices, food processing degree, gut microbiota, metabolomics, rats

## Abstract

The consumption of processed foods has increased compared to that of fresh foods in recent years, especially due to the coronavirus disease 2019 pandemic. Here, we evaluated the health effects of clarified apple juices (CAJs, devoid of pectin and additives) processed to different degrees, including not-from-concentrate (NFC) and from-concentrate (FC) CAJs. A 56-day experiment including a juice-switch after 28 days was designed. An integrated analysis of 16S rRNA sequencing and untargeted metabolomics of cecal content were performed. In addition, differences in the CAJs tested with respect to nutritional indices and composition of small-molecule compounds were analyzed. The NFC CAJ, which showed a higher phenolic content resulting from the lower processing degree, could improve microbiota diversity and influence its structure. It also reduced bile acid and bilirubin contents, as well as inhibited the microbial metabolism of tryptophan in the gut. However, we found that these effects diminished with time by performing experiment extension and undertaking juice-switching. Our study provides evidence regarding the health effects of processed foods that can potentially be applied to public health policy decision making. We believe that NFC juices with a lower processing degree could potentially be healthier than FC juice.

## 1. Introduction

According to the degree and purpose of processing, a novel food classification system called NOVA (meaning novel in Portuguese) was proposed in 2017, which divides processed foods into four categories: unprocessed or minimally processed foods, processed culinary ingredients, processed foods, and ultra-processed foods [1,2]. NOVA’s public health advice is that excessive intake of processed foods should be avoided in order to improve diet nutrition [2]. Many studies have also demonstrated the relationship between ultra-processed foods and non-communicable diseases, including diabetes, obesity, cardiovascular disease, coronary heart disease, and cerebrovascular diseases [3,4,5]. In-depth scientific research is now crucial to fully understanding the connection between the degree of food processing and public health [6].

The consumption of fresh fruits has decreased, while that of fruit juices has increased in recent years, especially since the outbreak of coronavirus disease 2019 (COVID-19) [7,8]. Apple juice is the most popular juice worldwide due to its flavor and taste. It has been reported that the administration of phenolics and pectin [9], as well as cloudy apple juice, could significantly regulate the gut microbiota. The effects of clarified apple juice (CAJ)—which is characterized by high energy density and the presence of food additives, as well as a lack of dietary fiber—on the gut microbiota remains unknown.

The gut microbiota is a complex microbial ecosystem that is essential for human health via a reciprocal relationship, and its important role can be explained by the correlation between composition and health status. However, it can also induce diseases, such as obesity, diabetes, and cardiovascular disease [10,11]. Research has suggested that gut microbiota disorders could be a mechanism explaining the correlation between processed foods and metabolic syndromes [4,12].

In this study, CAJs with different processing degrees, not-from-concentrate and from-concentrate (NFC and FC, respectively, pectin and additives eliminated), were selected. First, the effect of CAJs was demonstrated. Then, the effects of CAJs with different processing degrees were evaluated separately. In addition, a juice-switch experiment was performed. This study could demonstrate the comprehensive influence of different CAJs, which could provide additional evidence for the health effect of foods with different processing degrees and instructions for consumption behavior and public health policy designation.

## 2. Materials and Methods

### 2.1. CAJ

CAJs, including the unsterilized NFC and concentrated apple juices, from four factories located in Shaanxi and Shandong provinces in 2019 and 2020 were collected. After sampling, CAJs were frozen immediately, transported by cold chain to the laboratory, and stored at −20 °C. NFC includes juices processed by high-pressure processing (HPP) sterilization and pasteurization. For HPP, unsterilized NFC was added to a polyethylene terephthalate (PET) plastic bottle and treated at 550 MPa for 5 min (CQC30L-600, Beijing Suyuan Zhongtian technology limited company, Beijing, China). For pasteurization, unsterilized NFC was pasteurized at 85 °C for 15 s using an FT74 UHT/HTST processing system (Armfield, Ringwood, UK) and then added to PET bottles. For FC, concentrated apple juice was restored to the total solid sugar content before concentration (±0.1 °Bx), pasteurized under 85 °C for 15 s, and added to PET bottles. The 2019 samples from factories in Shaanxi were used for difference analysis and biomarker discovery, whereas the 2020 samples from one of the factories in Shaanxi were used for animal intervention.

### 2.2. Animal Intervention

**Figure 1:**
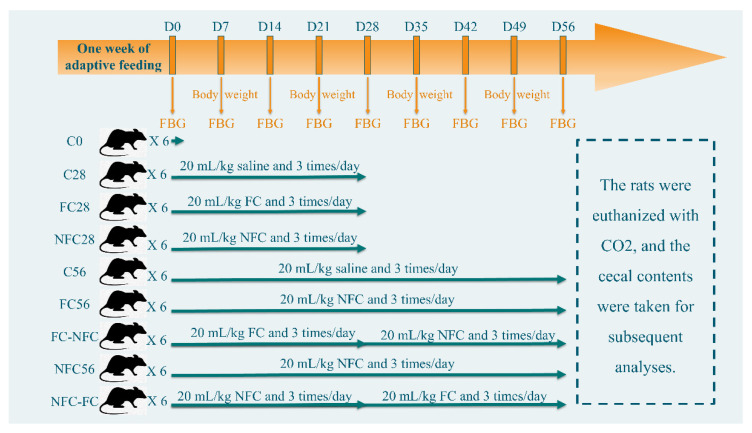
Schematic diagram of animal experimental scheme.

Fifty-four male Sprague Dawley rats aged 6–8 weeks provided by Charles River (Wilmington, MA, USA) were randomly and evenly divided into nine groups housed in 18 cages. The temperature of the animal facility was 20–23°C with a relative humidity of 45–60% and was under a 12 h dark–light cycle. The rats were free to drink and eat, and the main nutrients in the animal feed used in the experiment are provided in Supplemental Table S1. Food intake amount was calculated 3 times/week, with the total food weight loss of one cage divided by the number of rats in the cage. NFC (processed by HPP) and FC CAJs were used, and other detailed experimental designs are shown in Figure 1. The rats were gavaged 20 mL/kg body weight saline or FC or NFC 3 times per day. The gavage needle was slowly inserted into the mouth and then the esophagus. The juice was injected slowly to prevent returning to the mouth. Fasting glucose and plasma lipids were measured from blood from the eye socket after 12 h fasting and anesthesia by isoflurane.

### 2.3. 16 S rRNA Sequencing

Genomic DNA of the microbial community was extracted from the cecal content and analyzed using the Illumina MiSeq PE300 platform (Illumina, San Diego, CA, USA). Please refer to the online supplemental material for further details.

### 2.4. Untargeted Metabolomics

Metabolites extracted from the cecal content were analyzed using an AB SCIEX 6600 mass spectrometer coupled with ExionLC ultra-high-performance liquid chromatography (AB SCIEX, Framingham, MA, USA). Please see the online supplemental material methods section for further details.

### 2.5. CAJ Analyses

The glucose, fructose, sucrose, total phenol, amino acids, pH, and nutritional ingredients of the CAJs were analyzed. Table 1 presents the results. Furthermore, the small-molecule compounds were analyzed using UHPLC-QTOF. Please see the online supplemental material for details on the methods.

### 2.6. Data Processing, Statistical Analysis, and Visualization

For 16S rRNA sequencing, the data were analyzed with online tools from Majorbio (http://www.majorbio.com/, accessed on 25 July 2022). For metabolomic analyses, the peak extraction, alignment, and correction were performed using MS-DIAL ver. 4.36 software. MetaboAnalyst ver. 4.0 and SIMCA 14.1 were used for data analysis. MS-FINDER ver. 3.50, MassHunter PCDL ver. B.07.00, and LibraryView^TM^ ver. 1.1 were used for compound identification. Please see the online supplemental material methods section for further details.

## 3. Results

### 3.1. Continuous Intake of CAJ Has Limited Effect on Body Weight and No Effect on the Intake Amount, Gut Microbiota, and Blood Lipids

**Figure 2:**
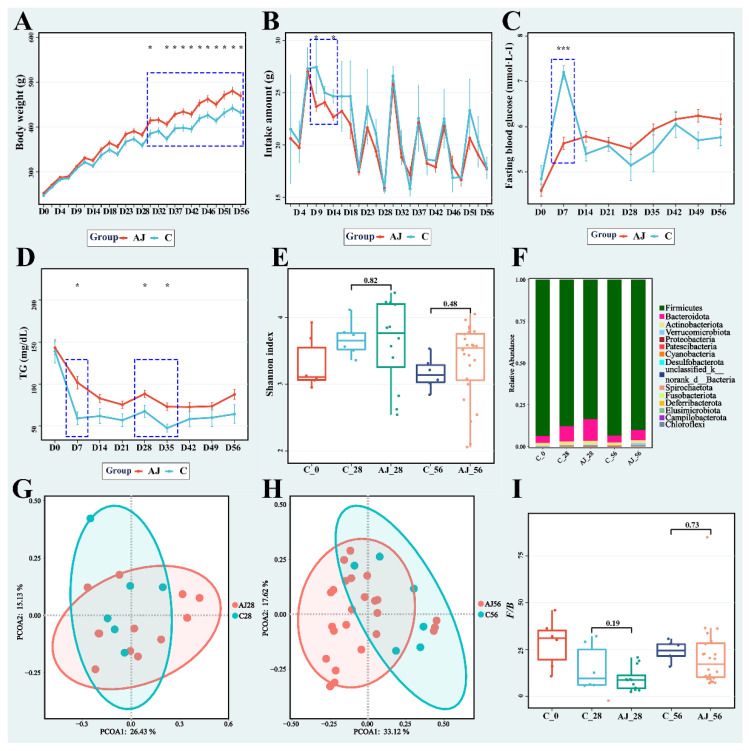
Effect of CAJ on rats: (**A**) Body weight. (**B**) Intake amount. (**C**) Fasting blood glucose. (**D**) Triglyceride content. (**E**) Shannon index. (**F**) Bar plot of gut microbiota on phylum level. (**G**) PCoA scores plot on OTU level of D28. (**H**) PCoA scores plot on OTU level of D56. (**I**) Box plot of *F*/*B* ratio. C, control group; AJ, apple juice groups, including NFC and FC groups. * indicates significant difference (*p* < 0.05) between CAJ and C groups. The “*” means *p* < 0.05, and “***” means *p* < 0.001.

As shown in Figure 2A, the body weight of the CAJ group was higher than that of the C group throughout the experimental period, and it was significantly different after D30 (*p* < 0.05, except for D32). However, the administration of fruit juice had no significant influence on the food intake amount (Figure 2B, where significance was only observed on D9 and D14). These results suggest that the energy intake of the CAJ group was approximately 24 kcal/kg body weight more per day (Supplemental Table S2). For fasting blood glucose (Figure 2C), no significant difference was observed except on D7 (*p* < 0.05). We also analyzed four parameters of blood lipids; the triglyceride levels (Figure 2D) in the CAJ group were always higher than those of the C group, but were significantly different only on D7, D28, and D35 (*p* < 0.05). The levels of the other three proteins (cholesterol, high-density lipoprotein, and low-density lipoprotein) are shown in Supplemental Figure S1. Only the high-density lipoprotein content of the C group on D7 was significantly higher than that of the AJ group (*p* < 0.05).

According to the *α*-diversity results shown in Figure 2E, no significant difference was observed between the groups at the same time point. Moreover, no obvious separation between the CAJ and C groups was observed in principal coordinate analysis (PCoA) score plots of *β*-diversity at the operational taxonomic unit (OTU) level on D28 and D56 (Figure 2G,H). In the gut microbiota at the phylum level, the dominant phyla were *Firmicutes* and *Bacteroidota*, whose relative abundance was higher than 80.8% in all samples (Figure 2F). According to numerous studies on gut microbiota and obesity, changes in the relative abundance of these two dominant bacteria, that is, an increase in *Firmicutes* and a decrease in *Bacteroidota*, can lead to an improvement in the host’s ability to obtain energy from foods, thus affecting the host’s energy balance and body weight [13]. This improvement may not immediately lead to obesity; however, a slight change in energy balance can cause significant changes in body weight over a long period [14,15]. Therefore, the ratio of *Firmicutes* to *Bacteroidota* could be a good biomarker for obesity [16]. As shown in Figure 2I, the ratio of the two dominant bacteria, *Firmicutes*/*Bacteroidota* (*F*/*B*), was not significantly different between groups at the same time point. Prolonged and continuous intake of CAJ may incrementally improve body weight, as revealed by the significantly higher body weight of the CAJ group. However, as the fasting blood glucose and *F*/*B* ratio were not significantly affected, and their energy intake was considerably higher, we conclude that the continuous intake of CAJ presents no health risk in rats.

### 3.2. CAJ with Lower Processing Degree Could Improve Microbiota Diversity and Inhibit the Metabolism of Bile Acids, Bilirubin, and Tryptophan in the Gut

**Figure 3:**
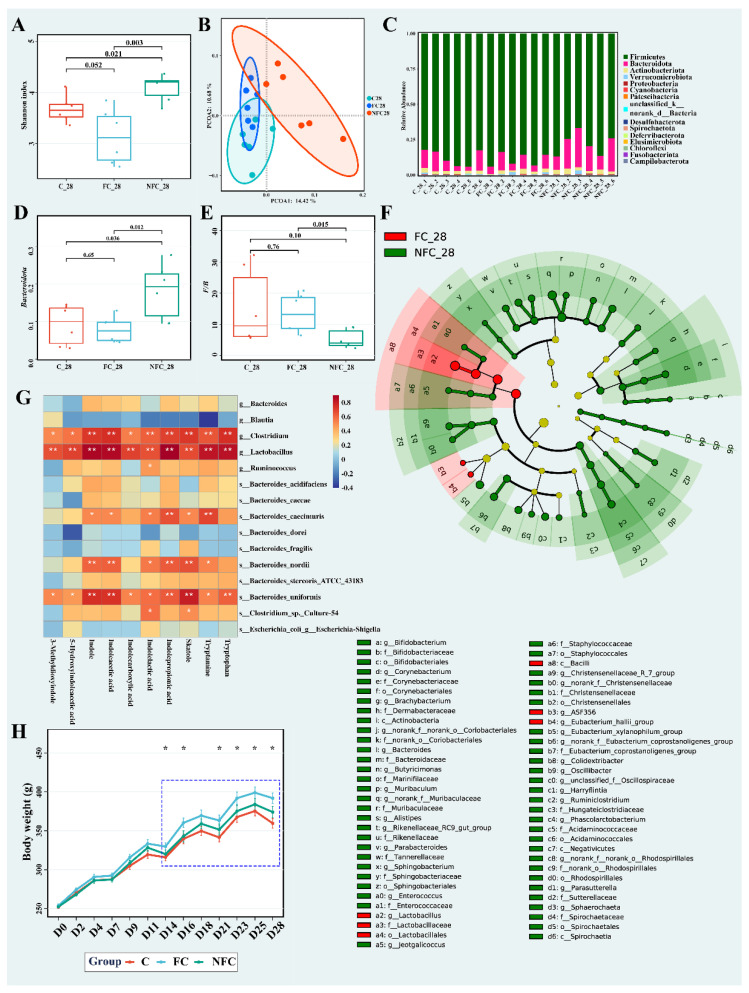
Effect of NFC and FC CAJs on rats: (**A**) Shannon index. (**B**) PCoA scores plot on OTU level. (**C**) Bar plot of gut microbiota on phylum level. (**D**) Box plot of *Bacteroidota* abundance. (**E**) Box plot of *F*/*B* ratio. (**F**) Cladogram from LEfSe analysis. (**G**) Correlation heatmap of microbiota and metabolites (The “*” means *p* < 0.05, and “**” means *p* < 0.01). (**H**) Body weight; * indicates significant difference (*p* < 0.05) between FC and C groups.

First, we focused on the first 28 days of administration. As shown in Figure 3A, the Shannon index of the FC group was not significantly different (*p* > 0.05) from that of the C group. In contrast, the NFC group with a lower processing degree was significantly different (*p* < 0.05) from the other two groups. The results indicated that NFC significantly improved the diversity of the gut microbiota in rats after 28 days of administration. Obesity is often accompanied by a decrease in the diversity of the gut microbiota [17]. In view of the *β*-diversity at the OTU level shown in Figure 3B, the NFC group was distinctly separated from the FC and C groups, which indicated that NFC could affect the gut microbiota structure, while FC had no such effect.

To further understand this discrepancy, we analyzed the abundance at multiple levels. More specifically, 15 phyla were detected at the phylum level (Figure 3C). *Firmicutes* abundance in the NFC group was significantly lower than that in the FC and C groups (Supplemental Figure S2A). In contrast, *Bacteroidota* abundance (shown in Figure 3D) in the NFC group was significantly higher than that in the other two groups (*p* < 0.05). As shown in Figure 3E, the *F*/*B* ratio was significantly lower in the NFC group than in the FC group. These results indicate that NFC could affect the dominant bacteria in the gut, thus reducing the risk of obesity. *Spirochaetota* abundance in the NFC group was significantly higher than in the FC and C groups (*p* < 0.05, Supplemental Figure S2B), and there was no significant difference between the FC and C groups. *Spirochaetota*, whose relative abundance was less than 0.04% in all samples, was detected in many environmental samples, with an unnoted proportion of 54.8% [18]. Furthermore, it is rich in carbohydrate hydrolases and is mainly involved in cellulose degradation [19]. In addition, its abundance in the gut of Hadza people from Tanzania was significantly higher than that of people from developed coastal areas [20]. In order to find biomarkers among NFC and FC groups, LDA effect size (LEfSe) analysis was used. As shown in Figure 3F, the dominant bacteria in the NFC group were primarily composed of *p_Bacteroidota*, *p_Actinobacteriota*, and *p_Spirochaetota*, while the dominant bacteria in the FC group were mainly located in *c_Clostridia* and *o_Lachnospirales*.

The effect of NFC on microbiota could also cause metabolite changes in the gut. The results of differential metabolites analysis between the NFC and FC groups, as well as their related metabolites, are shown in Table 2. The abundance of bile acids in the FC group (including cholic acid, deoxycholic acid, chenodeoxycholic acid, taurocholic acid, 7-ketodeoxycholic acid, and 3-oxo-4,6-choladienoic acid) was higher than that in the NFC group and comparable to that in the C group. Although there was no significant difference, the same trend of lithocholic acid content was observed.

As shown in Table 2, the bilirubin content in the FC group was higher than that in the NFC group, whereas the levels in the C group were comparable to those in the NFC group. Bilirubin is an important component of the heme catabolic pathway, which can be reduced to urobilinoids and/or urobilinogens by the gut microbiota. Urobilinogens are then either deposited into the feces as bile pigments or reabsorbed into the hepatic portal circulation. Urobilinogens are taken up by the kidneys, oxidized to urobilin, and excreted in the urine [21]. Bilirubin reductase may be derived from *Clostridium ramosum*, *Clostridium perfringens*, *Clostridium difficile*, or *Bacteroides fragilis*. Bilirubin can reduce hepatic fat accumulation, and increased levels of unconjugated bilirubin in the plasma have been suggested as treatments for obesity and type 2 diabetes mellitus [21].

As shown in Table 2, tryptophan levels in the FC group were significantly higher than that in the NFC group. In addition, its related metabolites, indole and its derivatives, also showed higher abundance in the FC group. The indolecarboxylic acid, indolelactic acid, indolepropionic acid, and 3-methyldioxyindole content in the FC group were significantly higher than those in the NFC group. Tryptophan is an essential amino acid that must be supplemented in the diet [22]. Tryptophan accounted for 0.26% of the rat diet (Supplemental Table S1), and tryptophan levels in CAJs were low and not significantly different (*p* > 0.075, Supplemental Figure S6).

Figure 3G shows a correlation heatmap between the abundance of metabolites and bacteria related to tryptophan metabolism [23]. *Clostridium*, *Lactobacillus*, and *Bacteroides_uniformis* were positively correlated with all tryptophan-related metabolites, and *Lactobacillus* abundance in the NFC group was significantly lower than that in the FC group. *Clostridium* and *Bacteroides_uniformis* also showed decreasing trends. In addition, *Bacteroides_nordii* and *Bacteroides_caecimuris* were positively correlated with six metabolites, whereas *Clostridium*_sp._*cultural-54* was positively correlated with indolelactic acid and skatole, and *Ruminococcus* was positively correlated with only indolelactic acid. These results indicate that differences between NFC and FC juices affected tryptophan metabolism in the gut. *Clostridium*, *Lactobacillus*, and *Ruminococcus* can convert tryptophan to tryptamine via tryptophan decarboxylase [23].

All rats displayed a continual increase in body weight during the experimental period, and there was no significant difference between the NFC and C groups at the same time point (Figure 3H). However, the body weight of the FC group was significantly higher than that of the C group after D14, except on D18 (*p* < 0.05). The weight of the FC group was the highest where the upward trend was the most evident.

### 3.3. Latter Intervention of NFC Did Not Show the Same Effect

**Figure 4:**
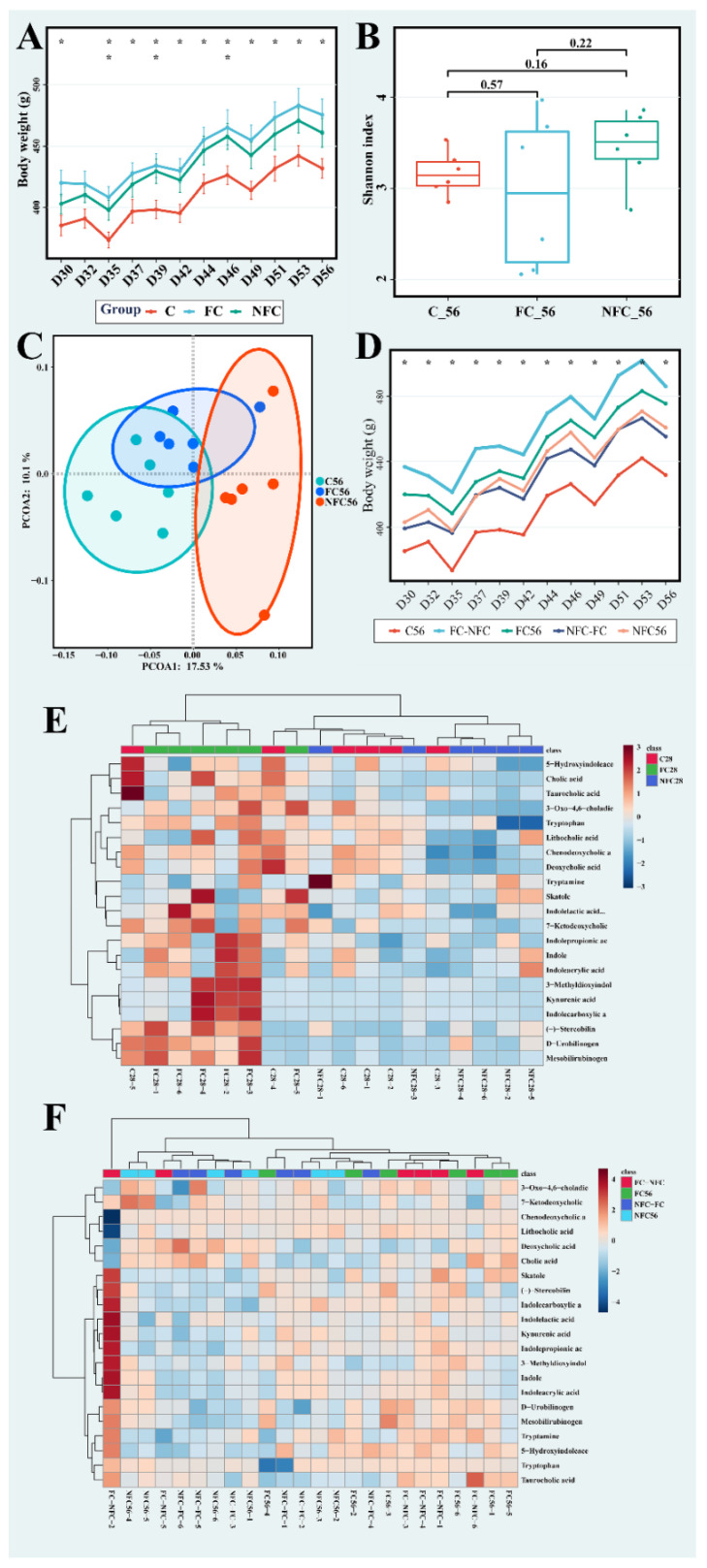
The effect of NFC and FC CAJ in rats in the prolonged and juice-switch period: (**A**) Body weight in prolonged period; * (*p* < 0.05) and ** (*p* < 0.01) in the first/second indicates significant difference between FC56/NFC56 and C56 groups. (**B**) Shannon index. (**C**) PCoA scores plot on OTU level. (**D**) Body weight in juice-switch period; * indicates the significant difference (*p* < 0.05) between FC-NFC and C56 groups. (**E**,**F**) Heatmaps from hierarchical clustering analysis for differential metabolites on D28 and D56.

To observe the long-term effects of NFC and FC, we extended the experimental period to 56 days. As shown in Figure 4A, body weight was not significantly different between the NFC and FC groups during the entire prolonged period. At D35, D39, and D46, body weight of the NFC group was significantly higher than that in the C group, while in almost all the days except D32, the weight of the FC group was significantly higher than that of the C group (*p* < 0.05). For the Shannon index (Figure 4B), there was no significant difference between the three groups. As for *Firmicutes*, *Bacteroidota*, *Spirochaetota*, and *F*/*B* shown in Supplemental Figure S7, no significant difference was observed between the NFC and FC groups. For *β*-diversity, there was no distinct separation between the NFC and FC groups in the PCoA scores plot (Figure 4C). Based on the above results, as the experimental period was prolonged, the effect of NFC compared to FC was diminished.

NFC could improve microbiota diversity and inhibit several intestinal metabolic processes in the former intervention, but we also observed that these beneficial effects would diminish over a prolonged period. We therefore designed the juice-switch groups. The rats that were treated with NFC were administered FC, and vice versa. The body weights of the juice-switch groups were similar to those before the juice-switch, as shown in Figure 4D. The NFC and NFC-FC groups had higher body weights compared to that of the C group, whereas weights in the FC and FC-NFC groups were much higher. The FC-NFC group was always significantly higher than the C group (*p* < 0.05). The Shannon index between the five groups showed no significant differences (Supplemental Figure S8). As for hierarchical clustering analysis based on differential metabolites (Figure 4E,F), the clustering of groups on D56 (including juice-switch groups) was not as obvious as groups on D28 (Figure 4E).

### 3.4. The Beneficial Effect of NFC Could Come from Polyphenol Compounds

In the present study, we analyzed the basic indices and nutritional ingredients of CAJ used in animal experiments. As shown in Table 1, the total phenol content of NFC juice was more than two times higher than that of FC samples, which indicated the mass loss of phenols during the concentration process. However, the nutritional ingredients, such as energy, remained the same (Supplemental Table S2).

Furthermore, we also analyzed the small-molecule compounds (including all the samples collected, not only the samples used for animal experiments) and determined the differences between them. It was observed from the PCA score plots in Supplemental Figure S3 that CAJs with different processing methods were distinctly separated, indicating a significant difference between them. Simultaneously, we observed that samples from the same processing method were clustered into multiple clusters based on differences in origin. However, the differences resulting from origin were no more than those from the processing method. The corresponding heatmaps from the hierarchical clustering analysis also indicated similar results (Supplemental Figure S3). Student’s *t*-test, fold change, and orthogonal projection to latent structures discriminant analysis (OPLS-DA) were conducted to identify the latent constant differential compounds. A compound could be defined as characteristic based on the following parameters: *p* < 0.05, ≥2-fold change, and variable importance for projection (VIP, from OPLS-DA) values > 1. Therefore, compounds were annotated and identified using chemical standards. The results for 18 phenolic compounds, whose contents in NFC were significantly higher than those in FC, are shown in Table 3. The beneficial effects of NFC could be attributed to phenolic compounds.

## 4. Discussion

This study was conducted to evaluate the effects of CAJs (thus eliminating pectin), including NFC and FC, on the gut microbiota of rats. This research suggests that substances in fruit juices other than pectin could have a significant effect. FC, which was treated using enzymatic hydrolysis for pectin, thermal concentration, and restoration, could be classified as an ultra-processed food based on the NOVA classification system [1]. Studies have shown that ultra-processed foods reduce the diversity of the gut microbiota and disrupt microbial functions, further affecting the health status of the host [4,12]. Meanwhile, thermal processing of food leads to the destruction of heat-sensitive vitamins and phytochemicals or generation of harmful substances [12], which will further affect the community characteristics of the gut microbiota and reduce its diversity [24].

In our study, although with limited health effects, CAJ intake over a long-term period significantly increased the body weight of rats (shown in Figure 2A). However, there was no distinct difference in gut microbiota diversity between the CAJ and control groups at the same time point (shown in Figure 2E). Furthermore, body weight, microbiota, and metabolomics of cecal content were affected by the administration of CAJ from different processing degrees in the first 28 days. NFC could affect the microbiome structure, significantly increase *α*-diversity and *Bacteroidetes* abundance, and reduce *Firmicutes* abundance and the *F*/*B* ratio (shown in Figure 3A,D,E). In *β*-diversity analysis shown in Figure 3B, a distinct separation was observed between the NFC and FC groups. Meanwhile, NFC reduced the levels of bile acids, tryptophan, bilirubin, and their related metabolites in the gut (shown in Table 2). In addition, we analyzed and compared the basic indices and small-molecule compounds of NFC and FC, showing that the multi-polyphenol content (18 phenolic compounds in Table 3) and total phenolic content (in Table 1) in the NFC group were both two times higher than those in the FC group.

As reported, only 5–10% of phenolic compounds ingested into the body through diet can be directly absorbed in the small intestine, while the vast majority (90–95%) arrive in the gut and play a role through decomposition and metabolism by microorganisms [25]. Meanwhile, apple phenolic extract can inhibit inflammatory pathway activation, protect intestinal mucosa integrity, restore the disorder of bile acid metabolism, and improve the diversity of gut microbiota. Among the polyphenols observed as differential ones between NFC and FC, most can change gut microbiota abundance and regulate bacterial structure and inflammation, obesity, and energy metabolism, thus improving health status. In mice with colitis, phloretin can reduce *Firmicutes* abundance and improve *Bacteroidota* abundance to achieve bacterial community rebalancing [26]. Quercetin can increase bacterial diversity, reduce the *F*/*B* ratio, and restore bacterial imbalance caused by dextran sodium sulfate [27]. It was reported that catechin can improve *Bacteroidetes* abundance in obese rats [28]. Chlorogenic acid can reduce plasma lipids, reverse obesity and metabolic disorders induced by high-carbohydrate and high-fat diets, as well as improve microbiota diversity [29,30]. Moreover, it can prevent type 2 diabetes by affecting glucose absorption and carbohydrate metabolism [31]. Phlorizin can significantly reduce energy intake, body weight gain, fasting blood glucose, triglyceride and total cholesterol levels and improve fecal microbial diversity [32]. Caffeic acid can significantly improve obesity induced by a high-fat diet, promote lipid metabolism, reduce body weight and fat accumulation, improve lipid structure, increase energy consumption, restore gut microbiota imbalance, and increase the abundance of anti-obesity-related and butyrate-producing bacteria [33].

Bile acids are synthesized from cholesterol by liver cells, stored in the gallbladder, and then released into the gut, which can promote the absorption of dietary fat and vitamins, as well as regulate glucose metabolism, lipid metabolism, energy homeostasis, etc. [34,35,36]. The composition of bile acids is closely related to obesity and is affected by gut microbiota [36]. As such, disturbance of the gut microbiota can alter the composition of bile acids, which may further alter important bile acid signaling pathways and affect host metabolism [36]. Unhealthy dietary habits (such as a long-term high-fat diet) increase the level of bile acids, aggravate the proliferation of stem cells, disrupt homeostasis in the gut, and may even lead to cancer [37]. The results shown in Table 2 indicated that NFC apple juice can reduce bile acid content and inhibit lipid absorption. Studies have shown that disorders of bile acid metabolism and the gut microbiota can increase intestinal permeability and aggravate the intestinal inflammatory response [38]. In addition, apple polyphenol extract can improve the intestinal inflammatory response by alleviating the disorder of bile acids and gut microbiota [38].

Tryptophan can be absorbed in the small intestine through food protein digestion and can enter blood circulation [39], while unabsorbed tryptophan travels to the large intestine and is broken down by bacteria to produce various indole derivatives, including indole, tryptamine, indoleethanol, indoleacetic acid, indolepropionic acid, indoleacrylic acid, indolealdehyde, and skatole [22]. Some bacteria in *Clostridium* and *Bacteroides* also metabolize tryptophan through tryptophanase. Indoleacetic acid can be generated by *Bacteroides* through indole-acetamide generated by tryptophan monooxygenase. *Lactobacillus* can also produce indole-3-lactic acid from aromatic amino acid aminotransferase and indolelactic acid dehydrogenase [23]. In our study, tryptophan and its related metabolite contents were found to be significantly correlated with tryptophan-metabolizing bacteria (shown in Figure 3G) [23].

Furthermore, there is a large amount of pectin in apple and cloudy apple juice, and some studies have found that these effects on gut microbiota could mainly be attributed to pectin [9]. The pectin contents of these two CAJs used were no more than 0.04 g/100 g because of the enzymatic hydrolysis process (Supplemental Table S2), which was far lower than the limit (5 g/100 g) at which it could have an obvious influence on gut microbiota [40]. Furthermore, the administration of pectin is usually accompanied with an increase in short-chain fatty acids; however, no such significant difference was observed in our results (Supplemental Figure S3).

During the prolonged experiment period, the effect diminished rather than accumulated (shown in Figure 4). This was not only indicated by the continuous administration of the same CAJ, but also by the juice-switch experiment. This may be because the intestinal sensitivity to phenolic compounds decreased as the rats aged. In addition, this effect could be counteracted by the sugar in CAJs. Sugar consumption is linked to an increase in obesity and non-communicable diseases; obesity is not the cause but rather a marker of metabolic dysfunction [41].

The results presented herein demonstrate the comprehensive effect of CAJ with different processing degrees. The evidence suggests that CAJ (with pectin eliminated) with a lower processing degree could lessen excessive increases in body weight by regulating the gut microbiota due to its higher multi-polyphenol contents. In a prolonged experiment, the combined effect of phenolics and pectin in cloudy apple juices may still be effective, while phenolics alone in CAJ could not counteract the effect of sugar. These results could be conducive to consumer behavior and public health policy design. Since the consumption of fruit juices has increased during the COVID-19 pandemic [7,8], more convenient and easy-to-transport fruit juices with a lower processing degree (with fewer additives and less processing) should be recommended.

## 5. Conclusions

In conclusion, NFC with higher phenolic content can significantly improve gut microbiota diversity and influence its structure. Simultaneously, it can reduce bile acids and bilirubin, as well as inhibit the microbial metabolism of tryptophan in the gut. However, these effects diminished with an extension of the experimental period. Furthermore, the juice-switch experiment confirmed that. The health outcomes and metabolomic differences between NFC and FC mainly originate from the phenolic differences caused by different processing methods and degrees. We believe that NFC juices that are processed with a lesser degree, although naturally containing a certain amount of sugar, could have more health benefits than we originally believed. In particular, with little attention paid to the total energy intake, NFC juices could be a viable option for consumption as natural polyphenol-rich foods.

## Figures and Tables

**Table 1:** Basic indices of NFC and FC.

Index	FC	NFC
Total phenol (GAE mg/100 mL)	1073.32 ± 72.33 ^b^	2169.03 ± 116.84 ^a^
pH	3.57 ± 0.02 ^b^	3.63 ± 0.02 ^a^
Sugar content (°Bx)	10.83 ± 1.41 ^a^	10.49 ± 0.11 ^a^
Glucose (g/100 mL)	7.81 ± 0.92 ^a^	6.13 ± 1.05 ^b^
Fructose (g/100 mL)	4.24 ± 0.79 ^a^	2.85 ± 0.74 ^b^
Sucrose (g/100 mL)	0.16 ± 0.06 ^a^	0.10 ± 0.02 ^a^
Malic acid (mg/100 mL)	186.16 ± 2.86 ^a^	187.10 ± 0.31 ^a^
Tartaric acid (mg/100 mL)	21.33 ± 0.08 ^a^	18.80 ± 0.15 ^b^
Asparagine (mg/100 mL)	4.57 ± 0.39 ^a^	10.82 ± 6.58 ^a^
Alanine (mg/100 mL)	4.43 ± 0.39 ^a^	10.73 ± 6.61 ^a^
Serine (mg/100 mL)	0.69 ± 0.09 ^a^	1.66 ± 1.08 ^a^
Arginine (mg/100 mL)	0.14 ± 0.02 ^a^	0.31 ± 0.19 ^a^
Glutamine (mg/100 mL)	0.07 ± 0.01 ^a^	0.15 ± 0.10 ^a^

GAE: gallic acid equivalent; Data marked with different letters indicate significant difference of *p* < 0.05; *n* = 3.

**Table 2:** Annotation results of differential and their related metabolites from cecal content.

Class or Name	Formula	C (%)	FC (%)	NFC (%)
**Bile acids**				
Cholic acid	C_24_H_40_O_5_	43.32 ^a^	47.92 ^a^	8.77 ^b^
Deoxycholic acid	C_24_H_40_O_4_	48.24 ^a^	38.52 ^a^	13.24 ^b^
Chenodeoxycholic acid	C_24_H_40_O_4_	41.40 ^a^	39.77 ^a^	18.83 ^b^
Taurocholic acid	C_26_H_45_NO_7_S	48.19 ^a^	37.35 ^a^	14.45 ^b^
Lithocholic acid	C_24_H_40_O_3_	35.38 ^a^	35.79 ^a^	28.83 ^a^
7-Ketodeoxycholic acid	C_24_H_38_O_5_	28.33 ^a,b^	49.52 ^a^	22.14 ^b^
3-Oxo-4,6-choladienoic acid	C_24_H_34_O_3_	34.36 ^a,b^	43.91 ^a^	21.73 ^b^
**Bilirubinoids**				
Mesobilirubinogen	C_33_H_44_N_4_O_6_	22.30 ^b^	60.23 ^a^	17.47 ^b^
D-Urobilinogen	C_33_H_42_N_4_O_6_	21.14 ^a^	57.88 ^a^	20.97 ^b^
(−)-Stercobilin	C_33_H_46_N_4_O_6_	20.85 ^b^	49.25 ^a^	29.90 ^b^
**Tryptophan and its metabolites**				
Tryptophan	C_11_H_12_N_2_O_2_	35.46 ^b^	43.08 ^a^	21.46 ^c^
Indole	C_8_H_7_N	29.30 ^b^	39.01 ^a^	31.69 ^a,b^
Tryptamine	C_10_H_12_N_2_	27.75 ^a^	29.21 ^a^	43.04 ^a^
Indolecarboxylic acid	C_9_H_7_NO_2_	13.77 ^b^	72.01 ^a^	14.22 ^b^
Indolelactic acid	C_10_H_9_NO_2_	34.41 ^a^	45.44 ^a^	20.15 ^b^
Indolepropionic acid	C_11_H_11_NO_2_	29.88 ^b^	42.13 ^a^	27.99 ^b^
Indoleacrylic acid	C_11_H_9_NO_2_	30.13 ^b^	38.90 ^a^	30.97 ^a,b^
Skatole	C_9_H_9_N	27.00 ^a^	39.77 ^a^	33.23 ^a^
3-Methyldioxyindole	C_9_H_9_NO_2_	13.77 ^b^	72.01 ^a^	14.22 ^b^
5-Hydroxyindoleacetic acid	C_10_H_9_NO_3_	49.36 ^a^	28.60 ^a,b^	22.05 ^b^
Kynurenic acid	C_10_H_7_NO_3_	18.87 ^b^	63.20 ^a^	17.94 ^b^

Last three columns show the relative percent contents between the three groups; Different letters indicate significant difference of *p* < 0.05; *n* = 6.

**Table 3:** Polyphenol compounds of NFC and FC.

Name	Fold Change (NFC/FC)	*p* Value	VIP
Chlorogenic acid	2.21	2.96 × 10^−7^	1.19
5-Methoxysalicylic acid	46.46	5.28 × 10^−17^	2.16
p-Coumaric acid	18.84	3.82 × 10^−12^	2.19
Caffeic acid	3.32	4.94 × 10^−6^	1.44
Ferulic acid	31.44	5.56 × 10^−14^	1.92
Phloretin	10.21	7.02 × 10^−7^	1.53
(+)-Catechin	16.76	1.71 × 10^−16^	2.24
(−)-Epicatechin	50.53	1.04 × 10^−16^	2.40
(−)-gallocatechin	2.92	7.55 × 10^−19^	1.85
Phlorizin	69.98	2.21 × 10^−16^	2.20
Isoquercitrin	59.83	3.96 × 10^−11^	2.09
Rutin	27.15	4.01 × 10^−12^	2.22
Naringenin	8.52	1.84 × 10^−9^	1.42
Eriodictyol	30.07	3.76 × 10^−12^	1.70
(+−)-Taxifolin	8.05	6.21 × 10^−13^	1.64
Quercetin-3-O-galactoside/Hyperoside	50.38	2.19 × 10^−12^	2.07
Procyanidin B1	102.51	2.84 × 10^−16^	2.51
p-Coumaraldehyde	67.65	2.98 × 10^−19^	2.90

VIP: variable importance for projection.

## Data Availability

The sequencing data of the 16S rRNA gene from this study are available in the Sequence Read Archive (SRA) under the project number PRJNA793573. Metabolomics data from mass spectrometry are available via MetaboLights with the identifier MTBLS4054.

## Supplementary Materials

The following supporting information can be downloaded at: https://www.mdpi.com/article/10.3390/nu14173488/s1. Figure S1: The cholesterol, high-density lipoprotein and low-density lipoprotein contents; Figure S2: The abundance of *Firmicutes* and *Spirochaetota* on D28; Figure S3: The PCA analysis of apple juices in positive and negative ion modes; Figure S4: The HCA analysis of apple juices in positive and negative ion modes; Figure S5: The OPLS-DA analysis of apple juices; Figure S6: The relative tryptophan contents in NFC and FC clarified apple juices; Figure S7: The abundance of *Firmicutes*, *Bacteroidota*, *Spirochaetota* and *F*/*B* on D56; Figure S8: Shannon index of five groups on D56; Table S1: The main nutrients of animal feed used in this experiment; Table S2: Nutrition ingredients of NFC and FC clarified apple juices; Table S3: The relative SCFA contents of D28 [42,43,44,45,46,47,48,49,50,51,52,53,54,55,56,57].
