# Supplementary material for: Impact of Clarified Apple Juices with Different Processing Methods on Gut Microbiota and Metabolomics of Rats
Source: Nutrients. 2022 Aug 25;14(17):3488. doi: 10.3390/nu14173488 (PMC9460580; doi:10.3390/nu14173488)
Supplement: Supplementary file 1 [file nutrients-14-03488-s001.zip › nutrients-1867281-supplementary.pdf]

Supplementary material

Impact of the degree of food processing on health outcomes: new evidence from gut microbiota and metabolomic analyses in rats fed clarified apple juices

Lei Xu,[1,2] **Shini Yang,**[1,2] **Kewen Wang,**[1,2] **Anjing Lu**[3,*]**, Xue Wang,**[1] **Zhenzhen Xu**[*,1]

[1] Institute of Quality Standard & Testing Technology for Agro-Products, Chinese Academy of Agricultural Sciences; Key Laboratory of Agro-food Safety and Quality, Ministry of Agriculture and Rural Affairs, Beijing 100081, China

[2] College of Food Science and Nutritional Engineering, China Agricultural University; Beijing Key Laboratory for Food Nonthermal Processing; Key Lab of Fruit and Vegetable Processing, Ministry of Agriculture and Rural Affairs, Beijing 100083, China

[3] China Center for Information Industry Development, Beijing 100048, China

[*]**Corresponding Author**

Tel: 861068205639; Fax: 861068205639; E-mail: luanjing@ccidthinktank.com

China Center for Information Industry Development, Ministry of Industry and Information Technology, Beijing 100036, P. R. China

Tel: 861082106560; Fax:861082106560; E-mail: xuzhenzhen@caas.cn

Institute of Quality Standard & Testing Technology for Agro-Products, Chinese Academy of Agricultural Sciences, Beijing 100081, P. R. China

Methods

Animal intervention

Table S1 The main nutrients of animal feed used in this experiment

Index	Content	Index	Content	Index	Content
Moisture (%)	8.00	Thr (%)	1.04	Folic acid (mg/kg)	11.90
Crude protein (%)	22.30	Leu (%)	2.36	Biotin (mg/kg)	0.40
Crude fat (%)	4.70	Ile (%)	1.23	VB12 (mg/kg)	0.04
Crude fiber (%)	3.50	Val (%)	1.46	Choline (mg/kg)	1900.00
Crude ash (%)	6.60	VA (KIU/kg)	19.00	Mg (%)	0.28
Ca (%)	1.18	VD (KIU/kg)	2.50	K (%)	0.95
P (%)	0.78	VE (IU/kg)	125.00	Na (%)	0.33
Lys (%)	1.72	VK (mg/kg)	10.00	Fe (mg/kg)	250.00
Met+Cys (%)	0.78	VB1 (mg/kg)	18.60	Mn (mg/kg)	130.00
Arg (%)	1.95	VB2 (mg/kg)	18.60	Cu (mg/kg)	19.90
His (%)	0.8	VB6 (mg/kg)	12.30	Zn (mg/kg)	67.00
Trp (%)	0.26	Nicotinic acid (mg/kg)	119.00	I (mg/kg)	1.00
Phe+Tyr (%)	2.23	Pantothenic acid (mg/kg)	30.00	Se (mg/kg)	0.20

Table S2 Nutrition ingredients of NFC and FC clarified apple juices

Index	FC	NFC
Energy (kcal/100g)	41.67	40.74
Moisture (g/100g)	89.39	89.65
Protein (g/100g)	0.12	0.13
Fat (g/100g)	ND	ND
Carbohydrate (g/100g)	10.29	10.07
Ash (g/100g)	0.15	0.13
Pectin (g/100g)	0.035	0.039
Na (mg/kg)	7.14	3.16
Ca (mg/kg)	39.25	36.85

Fasting glucose and plasma lipids: The blood from eye socket **was obtained** after 12 h fasting and anesthesia by isoflurane. Fasting glucose was measured with a glucometer (Roche, Basel, Switzerland). The blood were contributed (3500 *g*, 10 min) after 1 h standing to obtain the plasma. Then, the plasma lipids were measured with a blood biochemistry apparatus (C501, Roche, Basel, Switzerland).

16S rRNA sequencing

DNA extraction and PCR amplification: Microbial community genomic DNA was extracted from the samples using the DNA Kit (Omega Bio-tek, Norcross, GA, U.S.) according to the manufacturer's instructions. The DNA extract was checked on 1% agarose gel, and DNA concentration and purity were determined with NanoDrop 2000 UV-vis spectrophotometer (Thermo Scientific, Wilmington, USA). The hypervariable region V3-V4 of the bacterial 16S rRNA gene were amplified with primer pairs 338F (5'-ACTCCTACGGGAGGCAGCAG-3') and 806R(5'-GGACTACHVGGGTWTCTAAT-3') by an ABI GeneAmp® 9700 PCR thermocycler (ABI, CA, USA). The PCR amplification of 16S rRNA gene was performed as follows: initial denaturation at 95°C for 3 min, followed by 30 cycles of denaturing at 95°C for 30 s, annealing at 55°C for 30 s and extension at 72°C for 45 s, and single extension at 72°C for 10 min, and end at 4°C. The PCR mixtures contain 5 × *TransStart* FastPfu buffer 4 μL, 2.5 mM dNTPs 2 μL , forward primer (5 μM) 0.8 μL, reverse primer (5 μM) 0.8 μL, *TransStart* FastPfu DNA Polymerase 0.4 μL, BSA 0.2 μL, template DNA 10 ng, and finally ddH$_2$O up to 20 μL. PCR reactions were performed in triplicate. The PCR product was extracted from 2% agarose gel and purified using the AxyPrep DNA Gel Extraction Kit (Axygen Biosciences, Union City, CA, USA) according to the manufacturer's instructions and quantified using Quantus™ Fluorometer (Promega, USA).

Illumina MiSeq sequencing: Purified amplicons were pooled in equimolar and paired-end sequenced on an Illumina MiSeq PE300 platform (Illumina, San Diego,USA) according to the standard protocols by Majorbio Bio-Pharm Technology

Co. Ltd. (Shanghai, China).

Processing of sequencing data: The raw 16S rRNA gene sequencing reads were demultiplexed, quality-filtered by fastp version 0.20.0 [1] and merged by FLASH version 1.2.7 [2] with the following criteria: (i) the 300 bp reads were truncated at any site receiving an average quality score of <20 over a 50 bp sliding window, and the truncated reads shorter than 50 bp were discarded, reads containing ambiguous characters were also discarded; (ii) only overlapping sequences longer than 10 bp were assembled according to their overlapped sequence. The maximum mismatch ratio of overlap region is 0.2. Reads that could not be assembled were discarded; (iii) Samples were distinguished according to the barcode and primers, and the sequence direction was adjusted, exact barcode matching, 2 nucleotide mismatches in primer matching. Operational taxonomic units (OTUs) with 97% similarity cutoff [3, 4] were clustered using UPARSE version 7.1 [3], and chimeric sequences were identified and removed. The taxonomy of each OTU representative sequence was analyzed by RDP Classifier version 2.2 [5] against the Silva v138 16S rRNA database using confidence threshold of 0.7.

Untargeted metabolomics

Metabolite extraction: The method was previous published methods with some modifications.[6, 7] Thawed cecal content (300 mg) was mixed with 1.5 mL of pre-refrigerated methanol water (1:1 by volume) solution, and vortex for 30 s. The sample was frozen in liquid nitrogen for 30 s, and thawed in water at room temperature. This procedure was repeated for 3 times. Then, the sample was processed with ultrasound for 30 s, vortex for 10s, and rest for 30 s. This cycle was carried out for 5 times. Finally, the sample was centrifugated (at 4°C for 5 min with 15000 *g*) and filtered through a 0.22 μm filter.

Data acquisition: The sample was analyzed by the ultra-high performance liquid chromatography coupled to quadrupole time-of-flight mass spectrometry (UHPLC-QTOF, Exion LC-TripleTOF 6600, AB SCIEX, USA). The separation of compounds was conducted on a C18 column (C18, 1.7 μm, 2.1×100 mm, Waters, USA), maintained at 40°C. The mobile phases A and B were pure water and acetonitrile both with 0.1% formic acid. The gradient used was: 0.00-0.10 min, mobile phase B was maintained at 5%; 0.10-2.00, increased from 5% to 13%; 2.00-4.00, increased from 13% to 28%; 4.00-8.50, increased from 28% to 40%; 11.50-13.00, increased from 40% to 73%; 13.00-13.50, increased from 73% to 100%; 13.50- 16.00, maintained at 100%; 16.01-20.00, maintained at 5% for equilibration. The total flow rate was 0.300 mL/min, and the injection volume was 2 μL. The untargeted metabolomics analysis was conducted in positive and negative ion modes respectively using previous published methods with minor modifications.[8] The experiment parameters for MS1 scan were: curtain gas, 35; ion source gas 1, 50; ion source gas 2, 50; temperature, 500 °C; ion spray voltage floating, 5500 V for positive and -4500 V for negative ion mode; declustering potential, 80 V for positive and -80 V for negative ion mode; collision energy, 10 eV for positive and -10 V for negative ion mode; TOF MS scan *m/z* range was set as 50-1200 Da. Sequential Window Acquisition of All Theoretical

Fragment-ion (SWATH) mode was used. SWATH mode consists of a TOF MS (accumulation time, 0.05 s; collision energy, 10 eV for positive ion mode and -10 eV for negative ion mode) and 15 MS2 scans (accumulation time, 0.03 s for each; collision energy, 35 eV for positive ion mode, -35 eV for negative ion mode; collision energy spread, 15 eV). The SWATH window was optimized by the methods previous published.[8, 9] A quality control (QC) sample was prepared by mixing 5 μL of each sample. Three injections of the QC sample were initially conducted to equilibrate the column and the equipment, then the QC sample was injected regularly after every five injections of apple samples. The UHPLC-QTOF-MS data was acquired using Analyst TF software (Ver. 1.7.1, AB-SCIEX, USA).

Peak extraction, peak alignment and data correction

Peak extraction, peak alignment and peak table were performed for UHPLC-QTOF data by MS-DIAL Version 4.36 software. The parameters were set as follows: MS1 and MS2 tolerance were 0.01Da and 0.025Da respectively in peak extraction, and the maximum charge number was 2. MS1 and MS2 tolerance were 0.01 Da and 0.05 Da respectively in identification setting, and retention time deviation and MS1 deviation in peak alignment were 0.2 min and 0.015 Da. One peak with detection rate higher than 80% in at least one group, and S/N ratio (maximum in the sample/mean in the blank sample) higher than 3 was kept. Then, the aligned peak table was exported, and LOWESS (version 1.1) method was used to correct the response of each sample based on QC data and injection sequence, so as to eliminate the batch effect. After that, peak screening was carried out according to the detection rate and relative standard deviation (RSD) value of each peak in QC data, and the chromatographic peaks with detection rate less than 80% or RSD greater than 30% were removed.[10] The identification of metabolites of interest were conducted with MS-FINDER Version 3.50.

Clarified apple juice analyses

Glucose, fructose and sucrose: The methods were referred to published paper with some modifications.[11] After gradient dilution to 4000 times, the sample was filtered with a 0.22 μm filter, and then quantitatively analyzed by an ion chromatography. The column (PA20, 150 mm, inner diameter 3.0 mm) combining with a protective column (PA20, 30 mm, inner diameter 3.0 mm) were maintained at 30°C. Eluent A was pure water, and eluent B was 200 mmol/L sodium hydroxide solution. The flow rate was 0.4 mL/min, and the injection volume was 10 μL. The working electrode of the amperometric detector is Au electrode, and the reference electrode is Ag/AgCl. The temperature of the detection cell is 30°C. Gradient leaching conditions were as follows: 0~20 min, 5% B; 20.1-30 min, 20% B; 30.1~40 min, 100% B; 40.1~50 min, 5% B.

Total phenol content: The determination of total phenol content was referred to NY/T 1600-2008, and the result is expressed as Gallic acid equivalent (GAE) mg/100mL.

Amino acids: The determination of amino acids was referred to the published method.[12]

pH value: A small amount of apple juice was vortexed for 5 s, a calibrated pH meter was used to determine pH value at room temperature.

Nutrition ingredients: Energy calculation refers to GB/Z21922-2008 Basic terms of food nutrition; Moisture determination refers to GB 5009.3-2016; Protein determination was based on GB 5009.5-2016; Fat determination was based on the first method of GB 5009.6-2016; Carbohydrate was calculated by subtracting moisture, protein, fat and ash from total weight; ash determination refers to GB 5009.4-2016; Pectin determination refers to NY/T 2016-2011; Na and Ca were determined according to GB 5009.268-2016.

Small molecular compound analysis: Before analysis, the sample were centrifuged (15 min, 26916 *g*, 4 °C), filtered through a 0.45 μm and a 0.22 μm membrane filter in series. And the acquisition method was the same as the untargeted metabolomics analysis of cecal content. Then, PCA, T-test and differential multiple analysis were conducted using Metaboanalyst, and OPLS-DA model was constructed using SIMCA 14.1. Finally, the peaks, with $p < 0.05$, fold change > 2 and VIP value > 1, were defined as the potential markers. The MS2 of these potential markers were imported into MS-FINDER Version 3.50 software and searched with databases including Massbank, MONA, GNPS, HMDB, FooDB, RIKEN and locally built databases.[13-15] Meanwhile, the METLIN database was retrieved by MassHunter PCDL Version B.07.00.[16] Deviations for MS1 and MS2 are set as 10 ppm and 15 ppm, respectively. In order to confirm the compounds, the RT, MS1 and MS2 information of the standards were collected with chemical standards. Then, the databases of these information were constructed with LibraryView, and the data in the samples were retrieved by PeakView 2.2 to confirm these biomarkers. RT, MS1 and MS2 deviations were set to 0.2 min and 0.4 Da, respectively.

Results

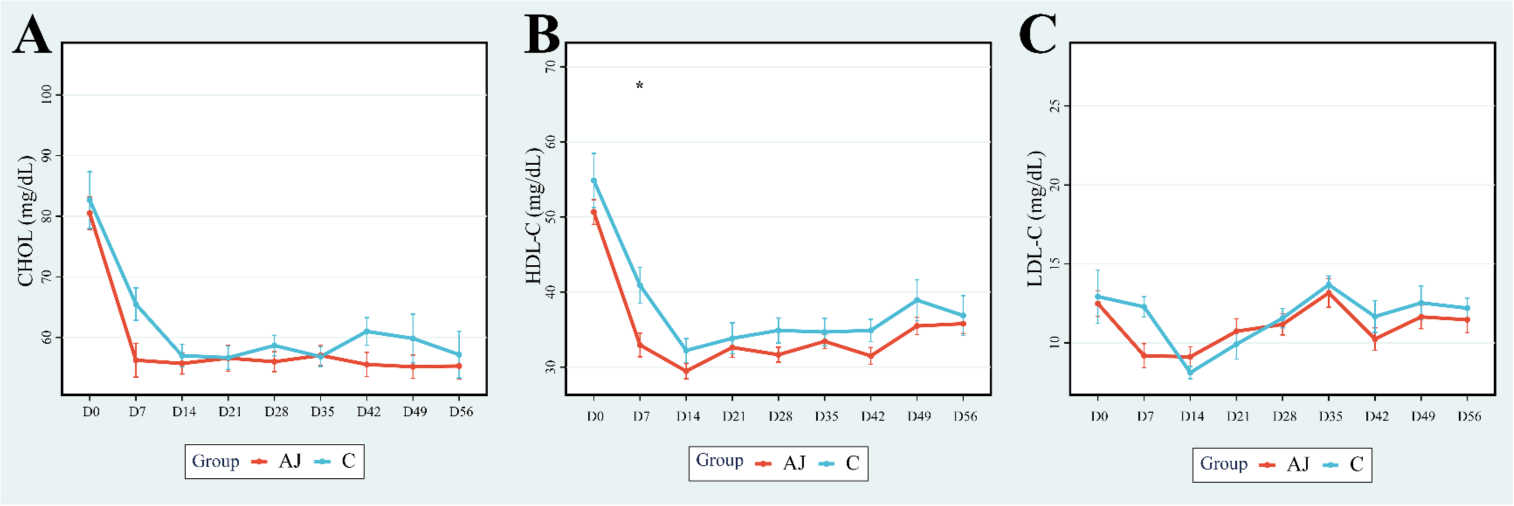

Figure S1 The cholesterol (A), high-density lipoprotein (B) and low-density lipoprotein (C) contents.

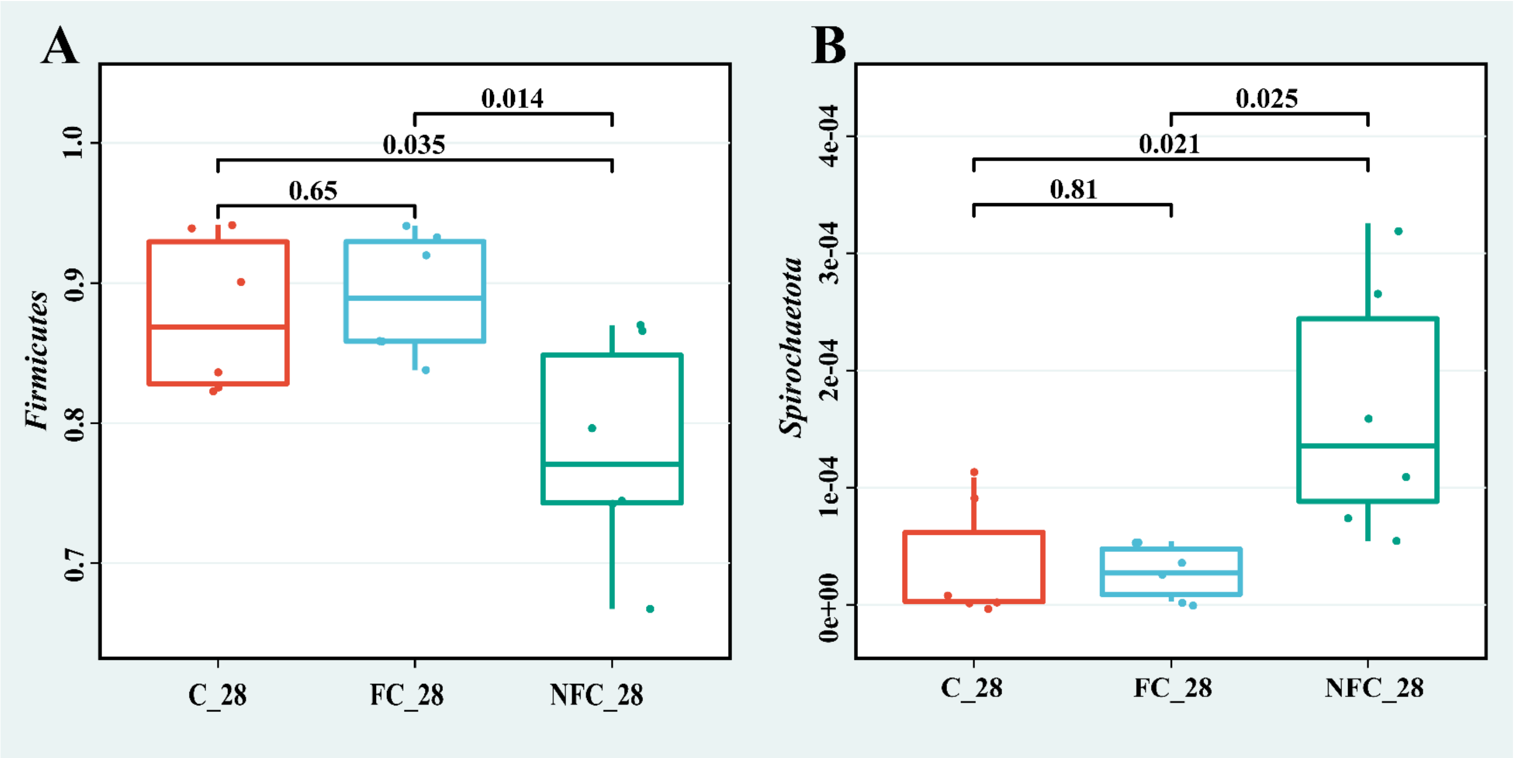

Figure S2 The abundance of *Firmicutes* (A) and *Spirochaetota* (B) on D28.

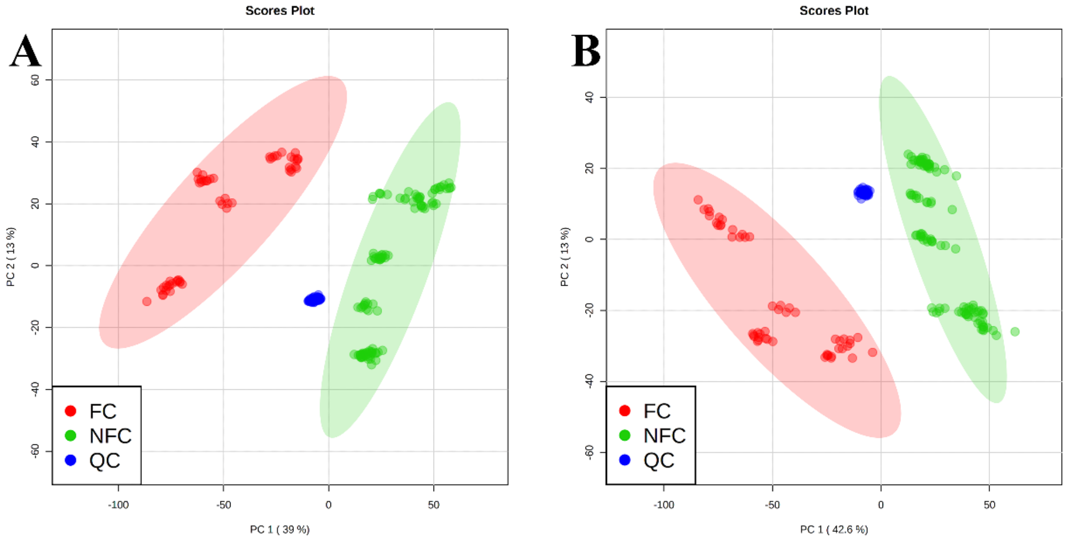

Figure S3 The PCA analysis of apple juices in positive (A) and negative ion modes (B).

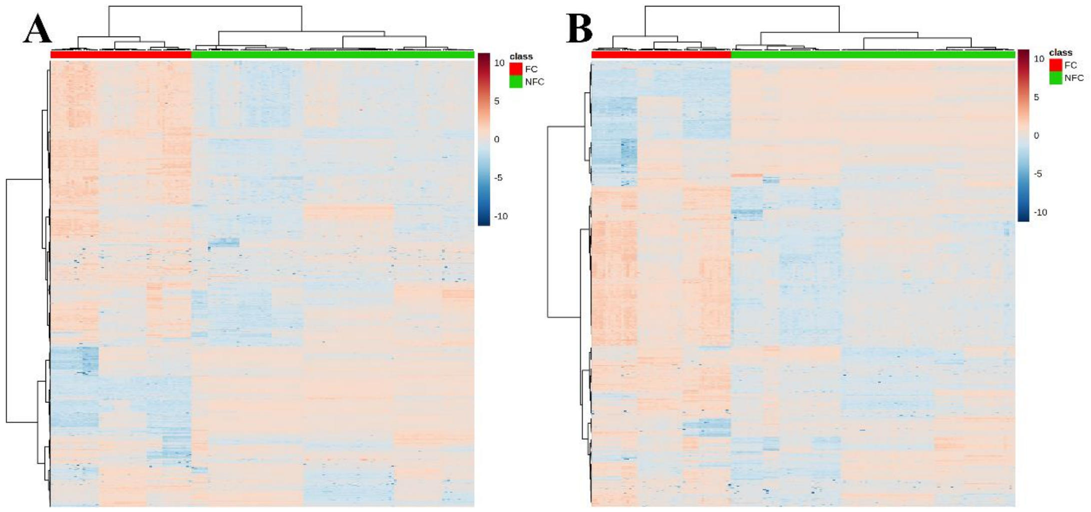

Figure S4 The HCA analysis of apple juices in positive (A) and negative ion modes (B).

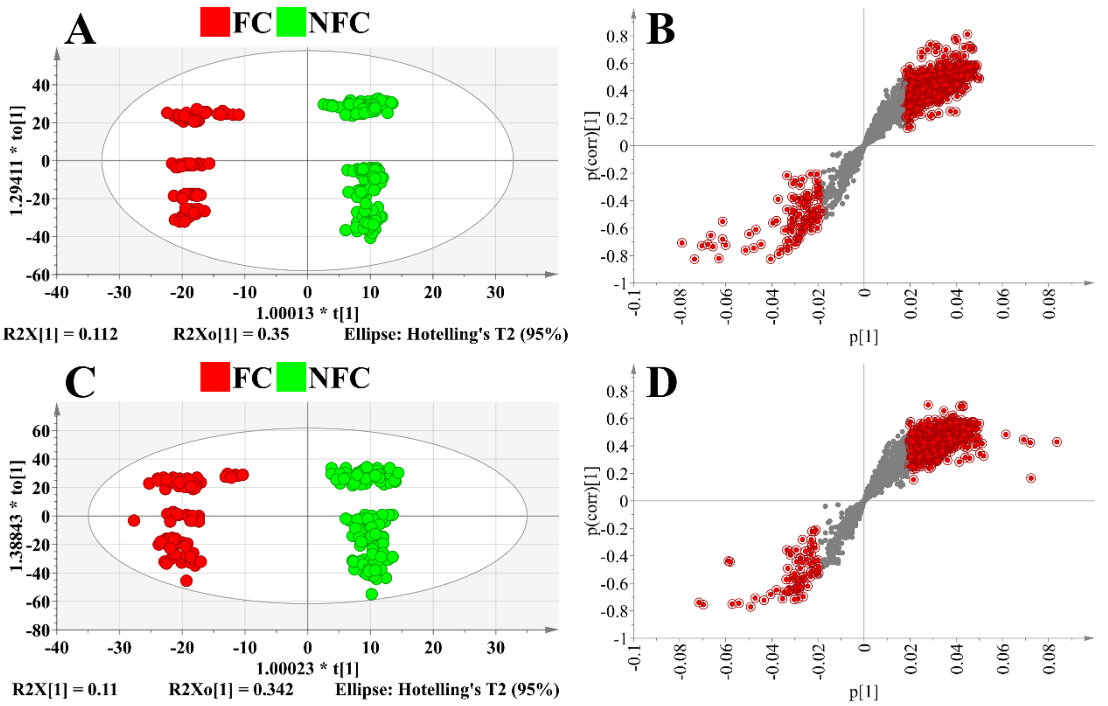

Figure S5 The OPLS-DA analysis of apple juices. (A) and (C), the scores plot in positive and negative ion modes, (B) and (D), the S-plots in positive and negative ion modes.

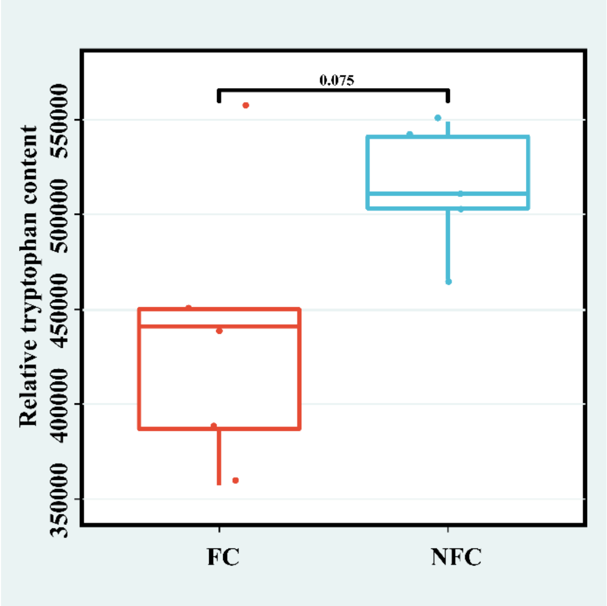

Figure S6 The relative tryptophan contents in NFC and FC clarified apple juices.

Table S3 The relative SCFA contents of D28

Sample Name	C28	FC28	NFC28
Acetate	107205±8515[a]	111475±18557[a]	113713±9818[a]
Propionic acid	169473±17973[a]	168937±36190[a]	166811±20752[a]
Butyric acid	652907±122875[a]	691654±118964[a]	610033±71686[a]
Valeric acid	57302±9537[a]	56348±16385[a]	52103±17209[a]

Isovaleric acid	11734±4377[a]	13594±1911[a]	12615±2762[a]
Hexanoic acid	93032±63204[a]	141402±89964[a]	145025±111906[a]

The same letter indicates no significant different.

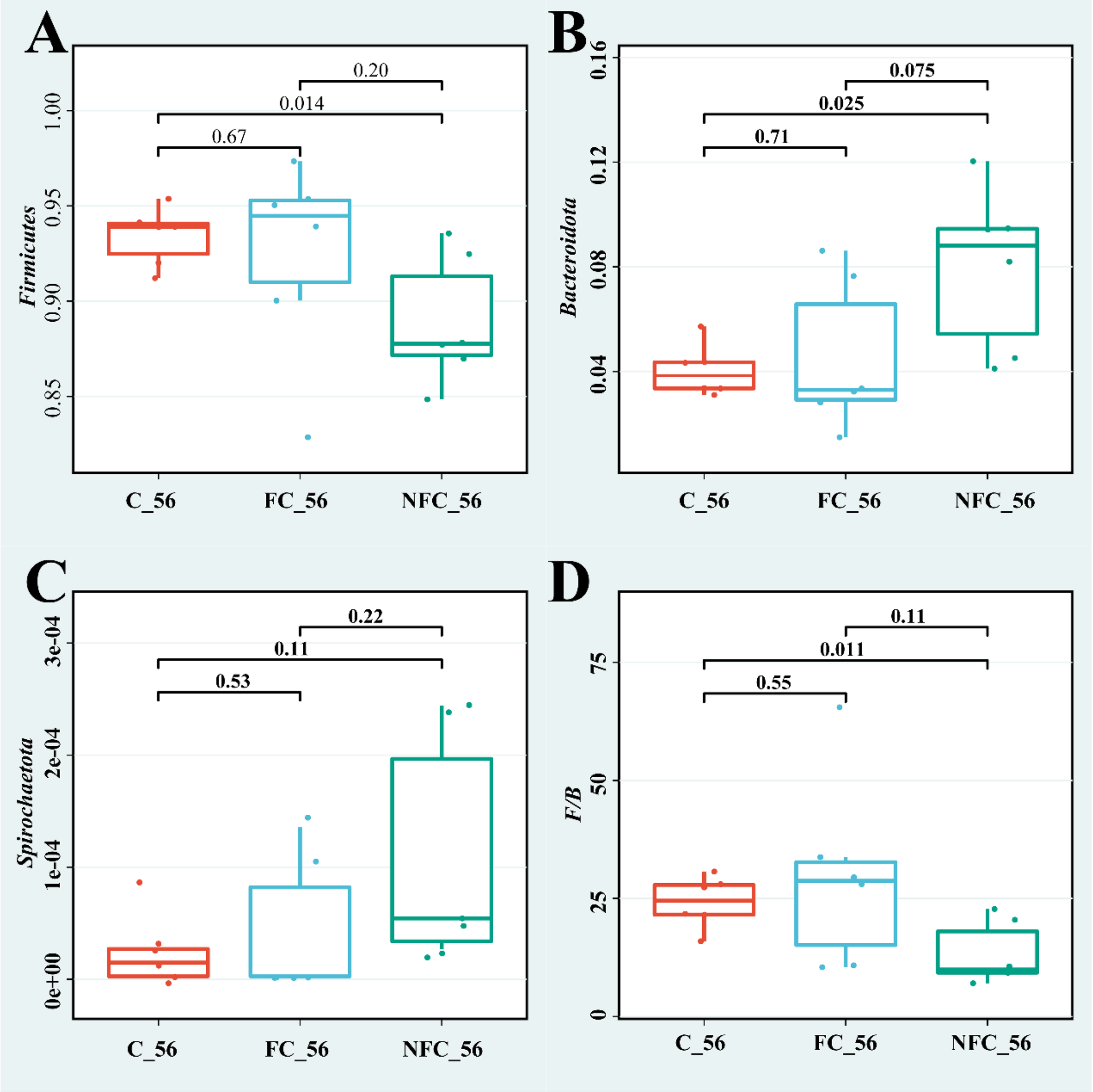

Figure S7 The abundance of *Firmicutes* (A), *Bacteroidota* (B), *Spirochaetota* (C) and *F/B* (D) on D56.

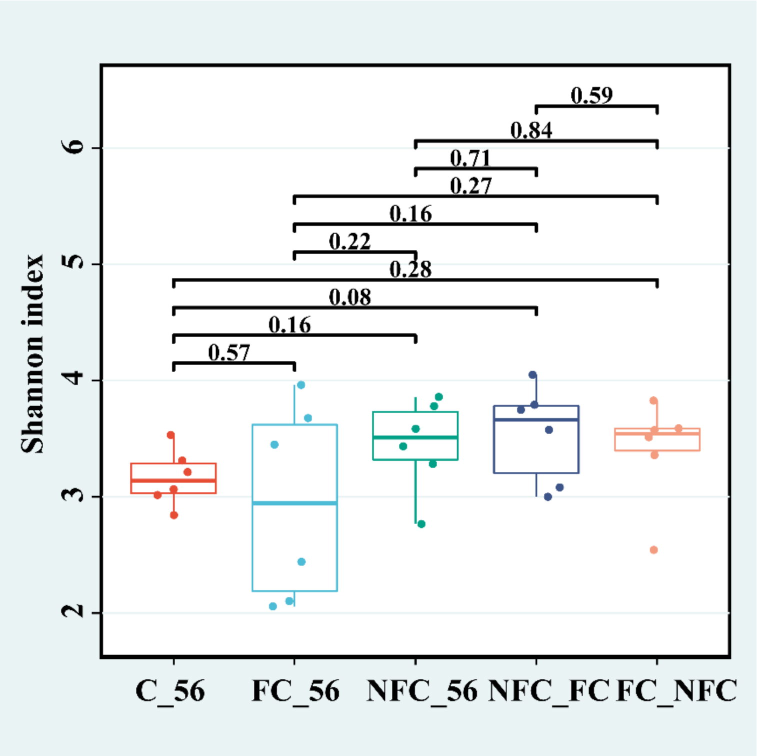

Figure S8 Shannon index of five groups on D56.

References

[1] S. Chen, Y. Zhou, Y. Chen, J. Gu, Bioinformatics. **2018**, 34, i884-i890.

[2] T. Magoc, S. L. Salzberg, Bioinformatics. **2011**, 27, 2957-2963.

[3] R. C. Edgar, Nature Methods. **2013**, 10, 996-998.

[4] E. Stackebrandt, B. M. Goebel, Int. j. syst. bacteriol. **1994**, 44, 846-849.

[5] Q. Wang, G. M. Garrity, J. M. Tiedje, J. R. Cole, Applied and Environmental Microbiology. **2007**, 73, 5261-5267.

[6] Y. Tian, L. Zhang, Y. Wang, H. Tang, Journal of Proteome Research. **2012**, 11, 1397-1411.

[7] G. Xie, W. Zhong, X. Zheng, Q. Li, Y. Qiu, H. Li, H. Chen, Z. Zhou, W. Jia, Journal of Proteome Research. **2013**, 12, 3297-3306.

[8] L. Xu, Z. Xu, I. Strashnov, X. Liao, Metabolomics. **2020**, 16, 81.

[9] Y. Zhang, A. Bilbao, T. Bruderer, J. Luban, C. Strambio-De-Castillia, F. Lisacek, G. Hopfgartner, E. Varesio, Journal of Proteome Research. **2015**, 14, 4359-4371.

[10] B. Sabina, B. Ivana, E. R. Verheij, R. Raymond, K. Sunil, I. A. Macdonald, V. O. Ben, A. K. Smilde, Analytical Chemistry. **2006**, 78, 567-574.

[11] B. Wang, X. Wang, J. Bei, L. Xu, X. Zhang, Z. Xu, Food Analytical Methods. **2021**, 14, 1227-1238.

[12] S. Wei-de, D. Li-jun, G. Yao-hua, Z. Chuan-jie, Y. Xiang-ying, S. Ming-qin, China Food Additives. **2019**, 30, 132-138.

[13] Z. Lai, H. Tsugawa, G. Wohlgemuth, S. Mehta, M. Mueller, Y. Zheng, A. Ogiwara, J. Meissen, M. Showalter, K. Takeuchi, T. Kind, P. Beal, M. Arita, O. Fiehn, Nature Methods. **2018**, 15, 53-56.

[14] H. Tsugawa, T. Kind, R. Nakabayashi, D. Yukihira, W. Tanaka, T. Cajka, K. Saito, O. Fiehn, M. Arita, Analytical Chemistry. **2016**, 88, 7946-7958.

[15] G. Wohlgemuth, S. S. Mehta, R. F. Mejia, S. Neumann, D. Pedrosa, T. Pluskal, E. L. Schymanski, E. L. Willighagen, M. Wilson, D. S. Wishart, M. Arita, P. C. Dorrestein, N. Bandeira, M. Wang, T. Schulze, R. M. Salek, C. Steinbeck, V. C. Nainala, R. Mistrik, T. Nishioka, O. Fiehn, Nature Biotechnology. **2016**, 34, 1099-1101.

[16] R. Tautenhahn, K. Cho, W. Uritboonthai, Z. Zhu, G. J. Patti, G. Siuzdak, Nature Biotechnology. **2012**, 30, 826-828.
